# Supplementary material for: Neuroprotective Potential of L-Glutamate Transporters in Human Induced Pluripotent Stem Cell-Derived Neural Cells against Excitotoxicity
Source: Int J Mol Sci. 2023 Aug 9;24(16):12605. doi: 10.3390/ijms241612605 (PMC10454411; doi:10.3390/ijms241612605)
Supplement: Supplementary file 1 [file ijms-24-12605-s001.zip › ijms-2431821-supplementary.pdf]

Blocker	Activity	IC_{50} EAAT1	EAAT2	EAAT3	Concentration used in this study	Ref
**TFB-TBOA**	**Broad** long lasting	22 nM	17 nM	300 nM	30 nM	[1]
**UCPH-101**	**Selective EAAT1** long lasting allosteric noncompetitive	0.66 μM	> 300 μM	> 300 μM	100 μM	[2]
**DHK**	**Selective EAAT2** competitive	> 3 mM	23 μM	> 3 mM	300 μM	[3]
**WAY213613**	**Potent EAAT2** competitive	5 μM	85 nM	3 μM	10 μM	[4]

IC_{50} values of blocker for the respective glutamate transporter and concentration used in this study.

1. Shimamoto, K.; Sakai, R.; Takaoka, K.; Yumoto, N.; Nakajima, T.; Amara, S.G.; Shigeri, Y. Characterization of novel L-threo-beta-benzyloxyaspartate derivatives, potent blockers of the glutamate transporters. *Mol Pharmacol* **2004**, *65*, 1008-1015, DOI:10.1124/mol.65.4.1008.
2. Abrahamsen, B.; Schneider, N.; Erichsen, M.N.; Huynh, T.H.; Fahlke, C.; Bunch, L.; Jensen, A.A. Allosteric modulation of an excitatory amino acid transporter: the subtype-selective inhibitor UCPH-101 exerts sustained inhibition of EAAT1 through an intramonomeric site in the trimerization domain. *J Neurosci* **2013**, *33*, 1068-1087, DOI:10.1523/jneurosci.3396-12.2013.
3. Arriza, J.L.; Fairman, W.A.; Wadiche, J.I.; Murdoch, G.H.; Kavanaugh, M.P.; Amara, S.G. Functional comparisons of three glutamate transporter subtypes cloned from human motor cortex. *J Neurosci* **1994**, *14*, 5559-5569.
4. Dunlop, J.; McIlvain, H.B.; Carrick, T.A.; Jow, B.; Lu, Q.; Kowal, D.; Lin, S.; Greenfield, A.; Grosanu, C.; Fan, K.; et al. Characterization of novel aryl-ether, biaryl, and fluorene aspartic acid and diaminopropionic acid analogs as potent inhibitors of the high-affinity glutamate transporter EAAT2. *Mol Pharmacol* **2005**, *68*, 974-982, DOI:10.1124/mol.105.012005.

Supplemental Table S1
